# Biomechanical Restoration Potential of Pentagalloyl Glucose after Arterial Extracellular Matrix Degeneration

**DOI:** 10.3390/bioengineering6030058

**Published:** 2019-07-03

**Authors:** Sourav S. Patnaik, Senol Piskin, Narasimha Rao Pillalamarri, Gabriela Romero, G. Patricia Escobar, Eugene Sprague, Ender A. Finol

**Affiliations:** 1 Department of Mechanical Engineering, The University of Texas at San Antonio, One UTSA Circle, San Antonio, TX 78249, USA; 2 Department of Mechanical Engineering, Koc University, Rumelifeneri Kampusu, Istanbul 34450, Turkey; 3 Chemical Engineering Program, Department of Biomedical Engineering, The University of Texas at San Antonio, San Antonio, TX 78249, USA; 4 Department of Medicine, University of Texas Health San Antonio, San Antonio, TX 78229, USA

**Keywords:** pentagalloyl glucose, aneurysm, enzyme, biomechanics, aorta

## Abstract

The objective of this study was to quantify pentagalloyl glucose (PGG) mediated biomechanical restoration of degenerated extracellular matrix (ECM). Planar biaxial tensile testing was performed for native (N), enzyme-treated (collagenase and elastase) (E), and PGG (P) treated porcine abdominal aorta specimens (n = 6 per group). An Ogden material model was fitted to the stress–strain data and finite element computational analyses of simulated native aorta and aneurysmal abdominal aorta were performed. The maximum tensile stress of the N group was higher than that in both E and P groups for both circumferential (43.78 ± 14.18 kPa vs. 10.03 ± 2.68 kPa vs. 13.85 ± 3.02 kPa; *p* = 0.0226) and longitudinal directions (33.89 ± 8.98 kPa vs. 9.04 ± 2.68 kPa vs. 14.69 ± 5.88 kPa; *p* = 0.0441). Tensile moduli in the circumferential direction was found to be in descending order as N > P > E (195.6 ± 58.72 kPa > 81.8 ± 22.76 kPa > 46.51 ± 15.04 kPa; *p* = 0.0314), whereas no significant differences were found in the longitudinal direction (*p* = 0.1607). PGG binds to the hydrophobic core of arterial tissues and the crosslinking of ECM fibers is one of the possible explanations for the recovery of biomechanical properties observed in this study. PGG is a beneficial polyphenol that can be potentially translated to clinical practice for preventing rupture of the aneurysmal arterial wall.

## 1. Introduction

The etiology of abdominal aortic aneurysm (AAA) development is believed to be multi-factorial, in that (i) the pathology is initiated at the molecular level (protease- and enzyme-related); (ii) it builds up to the tissue level through extracellular matrix (ECM) and structural changes; and (iii) it manifests as geometrical-, biomechanical-, and blood flow-related alterations in the abdominal aorta, resulting in rupture if left untreated [1,2,3]. Of the numerous etiological theories of AAA pathology, the degraded ECM theory is the widely accepted one, as human AAA specimens usually exhibit a reduction in elastin content and elastin crosslinking, and an increase in collagen crosslinking [4]. Increased elastase activity leads to disorganized and tortuous elastin fibers [5], which represents a compromised organization of load bearing proteins, resulting in reduced aortic elasticity [4,6], and further weakening of the aortic wall. With the deficiency in elastin, collagen dominates the ECM [7]. Disease progression is characterized by an increase in matrixmetalloproteinase (MMP) activity, which subsequently yields elevated wall stress and concomitantly higher wall stress to strength ratios [4,6,7,8]. In addition, most aneurysms exhibit an intraluminal thrombus (ILT), which is also a source of proteolytic activity [9], increased wall weakening [10], and a preferential site for rupture [11]. This multifaceted presentation of the disease makes the discovery of potential pharmacological targets a complex one (i.e., it has to consider the biological factors, the biomechanical environment, and the presence of ILT as a potential transport barrier).

Anti-inflammatory or matrix metalloproteinase inhibiting chemicals are the primary choice for stabilizing the aortic extracellular matrix (ECM). We envision the use of pentagalloyl glucose (PGG), a multifunctional polyphenol [12,13], as a potential pharmacological agent for AAA suppression [14]. PGG has been shown to bind to elastin and collagen, and stabilizes the ECM [4,5]. PGG has multiple phenolic hydroxyl groups that have high affinity towards the hydrophobic regions of the tissues [15] and can bind to proline-rich proteins such as elastin and collagen by surface adsorption mechanisms [16]. Isenberg and colleagues applied PGG periadventitially to the abdominal aorta of adult male Sprague–Dawley rats, previously exposed to CaCl_2_-mediated aortic elastin injury, and found that early inhibition of aneurysm and stabilization of elastin lamellae is possible [17]. Since then, multiple studies have investigated nanoparticle-based PGG delivery to the site of AAA [18,19]. Sinha et al. [20] reported that addition of PGG to rat aneurysmal smooth muscle cells increased lysyl oxidase production, enhanced elastin crosslinking, and assisted in lowering MMP-2 levels. Using a rat CaCl_2_ model of AAA, Thirugnanasambandam and colleagues showed that PGG was able to mitigate the inflammatory response, lower the MMP-2,9 levels, and prevent biomechanical stress build up on the aortic wall [21]. PGG has been applied as a preventive measure in porcine AAA models by Kloster and co-authors [22]; they found that PGG was able to lower the abnormal dilation of the abdominal aorta to a certain extent. 

AAA porcine models based on elastase–collagenase combination are uncommon [23,24,25,26,27], but their combined effect produces maximal damage to the ECM and pronounced inflammatory infiltration in vivo. Conversely, in vitro studies utilizing enzymatic digestion of porcine aortic tissues have been reported widely [28,29,30,31,32,33]. The objective of the present work is to quantify biomechanical changes in ex-vivo porcine abdominal aortas after treatment with PGG. Biaxial mechanical testing was performed on native, elastase, and collagenase treated (to mimic the presence of AAA), and PGG-treated enzyme degraded porcine abdominal aorta specimens. We hypothesize that enzyme treated specimens will exhibit loss of biomechanical strength compared to native or PGG-treated tissue specimens. We report on the ability of PGG to restore the biomechanical properties of the porcine abdominal aorta after enzymatic damage (elastase + collagenase). 

## 2. Materials and Methods

### 2.1. Biomechanical Testing

**Figure 1:**
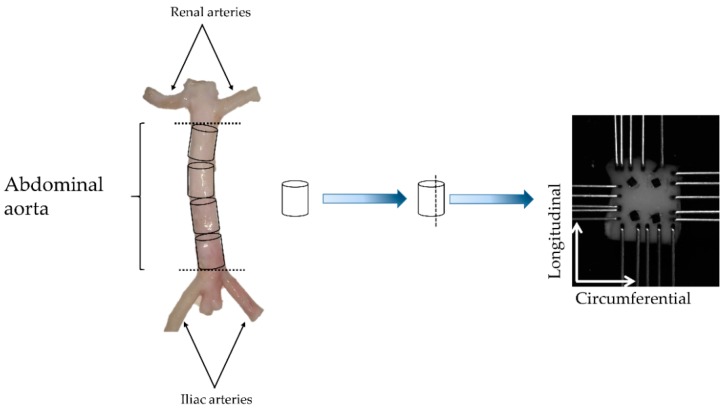
Exemplary schematic for specimen procurement. Each specimen consisted of a cylindrical ring approximately 7 mm long, which were subject to planar biaxial tensile testing. N = 6 specimens per group were tested using a CellScale BioTester^®^ while submerging the specimen in saline solution at 37 °C.

Three porcine abdominal aorta tracts (Yorkshire mixed breed, 125–250 lb, 6–9 months) were obtained from a local abattoir and all excess connective tissue removed. Approximately 7 mm-long cylindrical rings were dissected from the tracts and further utilized for biomechanical testing as shown in Figure 1. 

To evaluate the restorative potential of PGG, specimens were tested consecutively in their native state (N), followed by a simulated aneurysmal condition (E), and then treated with PGG (P). The simulated aneurysmal condition was achieved by treating the specimen for 1 h in an enzyme solution of 1.5 mg/mL purified elastase and 0.5 mg/mL purified collagenase at 37 °C [31] (Worthington Biochemical Corporation, Lakewood, NJ, USA). The treatment with PGG consisted of a 12-hour incubation in 0.6 mg/mL PGG at 4 °C after enzymatic treatment [22] (Sigma-Aldrich Inc., St. Louis, MO, USA). 

Specimens of an approximate size of 7 × 7 mm were prepared for biaxial testing as shown in Figure 1 and a suture knot (6-0 Silk—Ethicon Inc., Somerville, NJ, USA) was tied to the upper right-hand corner of the specimen to maintain the orientation throughout the study. Prior to testing, the wall thickness of each specimen was measured using digital caliper (Mitutoyo America Corporation, Aurora, IL, USA). Four fiducial markers were placed on the specimens using cyanoacrylate glue (Loctite Professional Super Glue Liquid, Henkel, Germany) for tracking local deformation, and the specimens were secured with metallic hooks as per the specified orientation (Figure 1). 

A CellScale© Biotester (CellScale Biomaterials Testing, Ontario, Canada) was utilized for biaxial mechanical testing of the specimens. Briefly, specimens were preloaded up to 2 g, preconditioned ten times in the physiological range (0–5% strain), and ultimately stretched equi-biaxially up to ~50% tensile strain over 45 seconds followed by unloading to its reference state [34,35,36]. Using a constant strain rate, the following strain-based protocol was performed ($\lambda_{C}:\lambda_{L}$)—1:1, 0.5:1, and 1:0.5—where $\lambda_{C}\;and\;\lambda_{L}$ represent stretch ratios in the circumferential and longitudinal directions, respectively. LabJoy 8.1 software (Waterloo Instruments Inc., Ontario, Canada) was utilized to collect the data at 30 Hz and images were captured at the rate of 1 Hz using the Biotester’s overhead CCD camera. All biomechanical tests were performed in a 37 °C saline bath. 

### 2.2. Data Analysis 

Force and displacement data were exported (LabJoy 8.1) and further processed to generate stress–strain curves for each specimen in the three experimental groups (N, E, and P) using MATLAB (R2018, The MathWorks Inc., Natick, MA, USA). Shear components of biaxial deformation were assumed negligible [37,38]. 

Stretch ratios in the circumferential and longitudinal directions are given by Equation (1),

(1) $$\lambda_{C^{\;}}=\;\frac{l_{C}}{l_{C0}},\;\lambda_{L^{\;}}=\;\frac{l_{L}}{l_{L0}},$$

where $l_{C0}$ and $l_{L0}$ are the undeformed specimen lengths in mm, and $l_{C}$ and $l_{L}$ are the deformed specimen lengths in mm, for the circumferential and longitudinal directions, respectively.

The Green strain tensor components were calculated from the respective stretch ratios following Equation (2),

(2) $$\epsilon_{C}=\frac{1}{2}\left(\lambda_{C}^{2}-1\right),\epsilon_{L}=\frac{1}{2}\left(\lambda_{L}^{2}-1\right)$$

where $\epsilon_{C}$ and $\epsilon_{L}$ are the Green strains in circumferential and longitudinal directions, respectively. 

The Cauchy stress (σ) was calculated by dividing the force by the cross-sectional area of each specimen (width multiplied by thickness), as indicated by Equation (3),

(3) $$\sigma_{C}=\;\frac{F_{C}*\lambda_{C^{\;}}\;}{A_{C}},\;\sigma_{L}=\;\frac{F_{L}*\lambda_{C^{\;}}}{A_{L}},$$

where $F_{C}$ and $F_{L}$ are forces in Newton, and $A_{C}$ and $A_{L}$ are the specimen cross-sectional areas in mm^2^, for the circumferential and longitudinal directions, respectively. 

**Figure 2:**
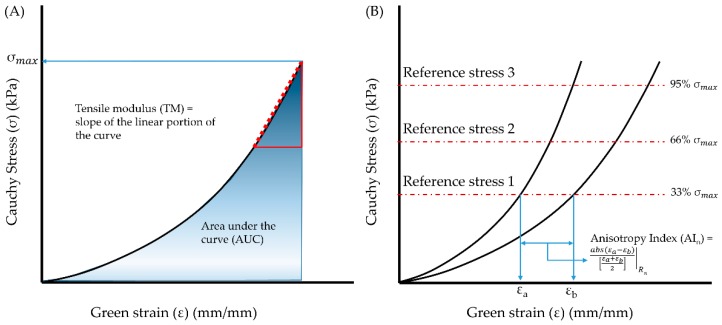
Biomechanical parameters evaluated from the stress–strain curves generated from biaxial mechanical testing of porcine abdominal aorta specimens. (**A**) Schematic for calculation of area under the curve (AUC) and maximum tensile stress ($\sigma_{max}$). (**B**) Procedure for calculation of anisotropy index (AI) at each reference stress level, as reported in [39,40]. The reference stresses for each specimen were calculated by estimating 33rd, 66th, and 95th percentiles of $\sigma_{max}$ in each stress–strain curve.

The tensile moduli (TM), defined as the slope of the upper linear portion of the stress–strain curves that best represents the linear elastic region of the material (see Figure 2A) for the circumferential and longitudinal directions, were calculated using a pointwise linear regression in the upper 10% of the strain range. The strain energy, determined by area under the stress–strain curve (AUC), and maximum stress ($\sigma_{max}$) were also calculated for both tissue orientations (see Figure 2A). 

Enzymatic damage introduces some degree of changes to the ECM and in many cases, a change in anisotropic behavior was observed in porcine arterial tissues [30]. To measure these changes in the mechanical anisotropy, we calculated the anisotropy index (AI, illustrated in Figure 2B) according to Equation (4) [39,40],

(4) $$Anisotropy\;Index\;\left(AI_{n}\right)\;=\;\frac{abs\left(\varepsilon_{a}-\varepsilon_{b}\right)}{\left[\frac{\varepsilon_{a}+\varepsilon_{b}}{2}\right]}|{\;}_{R_{n}}$$

AI was calculated for each group at reference stresses ($R_{n}$) corresponding to the 33rd, 66th, and 95th percentiles of the maximum stress ($\sigma_{max}$) in each orientation (i.e., $AI_{1}$, $AI_{2}$, and $AI_{3}$, respectively). A perfectly isotropic material will have an *AI* of zero, whereas tissues and most biological materials exhibit non-zero *AI* values. 

To quantify recovery, we normalized the biomechanical parameters obtained from the E and P groups to their N matching counterparts according to Equation (5),

(5) $$\mathrm{Normalized}\;\emptyset_{C\;\mathrm{or}\;L}\;=\;\frac{\;{\emptyset |}_{E\;}}{\;\emptyset_{N}};\;\frac{\;{\emptyset |}_{P}}{\;\emptyset_{N}}$$

where $\emptyset =\mathrm{biomechanical}\;\mathrm{parameter}\;\mathrm{such}\;\mathrm{as}\;\sigma_{max},\;\mathrm{TM},\mathrm{or}\;\mathrm{AUC}.$ C and L stands for circumferential or longitudinal. N, E, P are the three experimental groups. The aforementioned biomechanical data collected from the specimens were further utilized for constitute modeling and as input for finite element modeling. 

### 2.3. Constitutive Modeling

To characterize the material behavior of the specimens, a first-order incompressible hyperelastic Ogden material model [41,42,43], Equation (6), was fitted to the experimental stress–strain data,

(6) $$\Psi =\frac{m_{1}}{c_{1}}\;\left(\lambda_{1}^{c_{1}}+\lambda_{2}^{c_{1}}+\lambda_{3}^{c_{1}}-3\right)$$

where $c_{1}$ and $m_{1}$ are constants, and $\lambda_{i}$ are the principal stretches. For planar biaxial tension, there are no stretch data in the 3^rd^ direction. Therefore, $\lambda_{3}$ was obtained by applying an incompressibility condition (i.e., the determinant of F=1, where F is the deformation gradient tensor), as expressed by Equation (7).

(7) $$\lambda_{3}=\frac{1}{\lambda_{1}\cdot \lambda_{2}}$$

We have assumed that there are no shear components during the planar biaxial tension. The ANSYS (Ansys, Inc., Canonsburg, PA, USA) biaxial curve fitting tool (a non-linear least squares algorithm) was utilized to generate the best subset of material constants that can minimize the differences of the sum of the squares between the experimental data and the constitutive model. The tool uses the Levenberg–Marquardt algorithm to solve the non-linear least squares problem. This method requires a set of initial values for each parameter of the material model (i.e., $c_{1}$ and $m_{1}$), as many other optimization algorithms. The error calculation is performed using a normalized error instead of an absolute error. We calculated residual errors for all native and aneurysm samples, but do not report them in the manuscript since the models have an excellent goodness of fit, as described in Section 3.2. 

### 2.4. Finite Element Modeling

**Figure 3:**
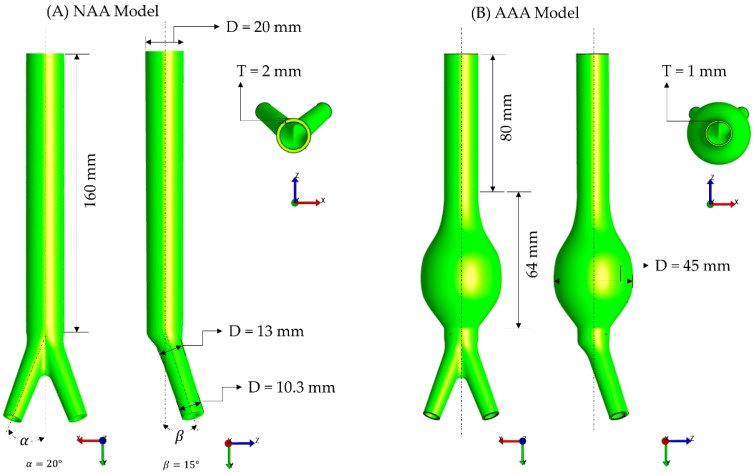
Geometric models and their dimensions for the (**A**) native abdominal aorta (NAA) and (**B**) aneurysmal abdominal aorta (AAA).

The material constants from the Ogden model fitting from each group (N, E, or P) were further utilized for computational modeling following previously established protocols [21,44]. Briefly, idealized models of a native abdominal aorta (NAA) and an aneurysmal abdominal aorta (AAA) were created using ANSYS^®^ SpaceClaim (SpaceClaim Corporation, Concord, MA, USA). Figure 3 shows the geometries of the models and their dimensions, based on the work reported by Azar et al. [44]. The inner and outer surfaces of the geometries were meshed with 2D triangle elements using Gmsh open source software [45]. The surface meshes were converted into volume meshes using TetGen [46] by generating linear tetrahedral elements. FEBio^®^ Preview was utilized to setup the volumetric meshes for both models [47]. The Ogden material properties obtained with the native tissue properties (N) were assigned to the NAA model, whereas the Ogden material properties obtained with the enzymatic (E) and PGG tissue properties (P) were assigned to the AAA model. Both ends of the models were fixed for all degrees of freedoms. An average of systolic and diastolic pressures (100 mmHg) was applied homogenously at the intraluminal surface of the models [21]. A quasi-static structural analysis was performed with the open source finite element analysis (FEA) solver FEBio [47]. The first principal stress [48] generated by the FEA simulations was postprocessed with FEBio PostView [47] to quantify the differences in in silico wall stress distributions due to changes in material properties (N group vs. E and P groups). 

### 2.5. Statistical Analysis

Data were reported as mean ± standard error of mean (SEM). The same porcine arterial specimens (N) underwent enzymatic treatment (E) and followed by PGG treatment (P), a repeated-measures ANOVA was performed to elucidate the differences across the biomechanical data ($\sigma_{max}$, *TM*, AI, and AUC). Sphericity was assumed for the data and pairwise comparisons were performed using Tukey’s test with results considered significant when *p* < 0.05. All analyses were performed using SPSS (IBM Corp., Armonk, NY, USA). 

## 3. Results

### 3.1. Biomechanical Testing

Biomechanical parameters such as $\sigma_{max}$ and AUC were found to be significantly different across the groups for both tissue orientations (*p* < 0.05), whereas TM was significantly different for only the circumferential direction. The anisotropy indices ($AI_{1}$, $AI_{2}$, and $AI_{3}$), derived from the three reference stresses, were found to be non-zero, but conserved across the three groups (*p* = 0.2702, *p* = 0.0813, and *p* = 0.1425, respectively). All biomechanical parameters calculated from the three biaxial testing protocols ($\lambda_{C}:\lambda_{L}$ – 1:1, 0.5:1, and 1:0.5) are listed in Table 1. 

**Figure 4:**
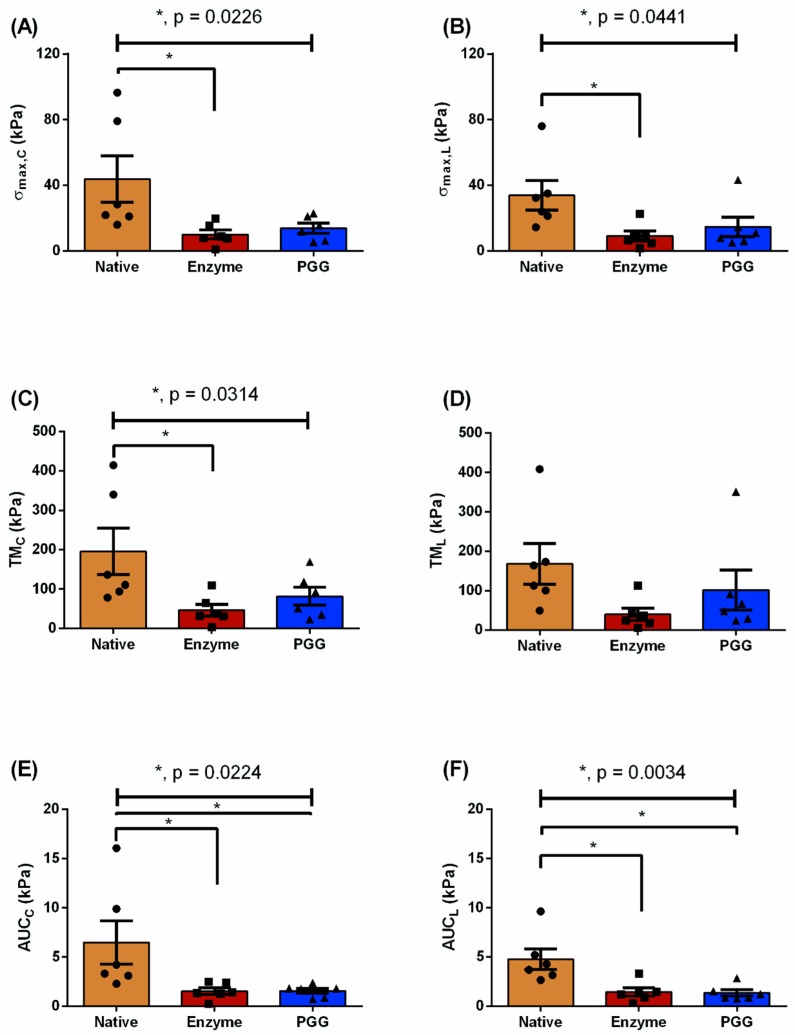
Biomechanical parameters for the native (N), elastase-digested (E), and PGG-treated (P) porcine abdominal aorta specimens, which are represented as mean ± SEM. Maximum stress (kPa) is displayed in both circumferential ($\sigma_{max,C}$) (**A**) and longitudinal orientations ($\sigma_{max,L}$) (**B**). (**C**,**D**) tensile moduli (TM) (kPa) and (**E**,**F**) area under the curve (AUC) (kPa) for both circumferential and longitudinal directions, respectively. *denotes significance across the groups (*p* < 0.05).

The maximum tensile stress of the N group was higher than in the E and P groups for both circumferential (43.78 ± 14.18 kPa vs. 10.03 ± 2.68 kPa vs. 13.85 ± 3.02 kPa; *p* = 0.0226) and longitudinal directions (33.89 ± 8.98 kPa vs. 9.04 ± 2.68 kPa vs. 14.69 ± 5.88 kPa; *p* = 0.0441), as shown in Figure 4A,B. Likewise, the tensile moduli was found to be in descending order as N > P > E for the circumferential direction (195.6 ± 58.72 kPa > 81.8 ± 22.76 kPa > 46.51 ± 15.04 kPa; *p* = 0.0314), as illustrated in Figure 4C, whereas no significant differences were found in the longitudinal direction (*p* = 0.1607). Strain energy, represented by AUC, was nearly four times greater for the N group than the E or P groups in the circumferential direction (6.48 ± 2.22 kPa vs. 1.55 ± 0.34 kPa or 1.56 ± 0.26 kPa; *p* = 0.0224), as shown in Figure 4E. For the longitudinal direction, AUC was nearly three times greater for the N group than the E or P groups (4.77 ± 1.04 kPa vs. 1.45 ± 0.42 kPa or 1.35 ± 0.32 kPa; *p* = 0.0034), as illustrated in Figure 4F. 

**Figure 5:**
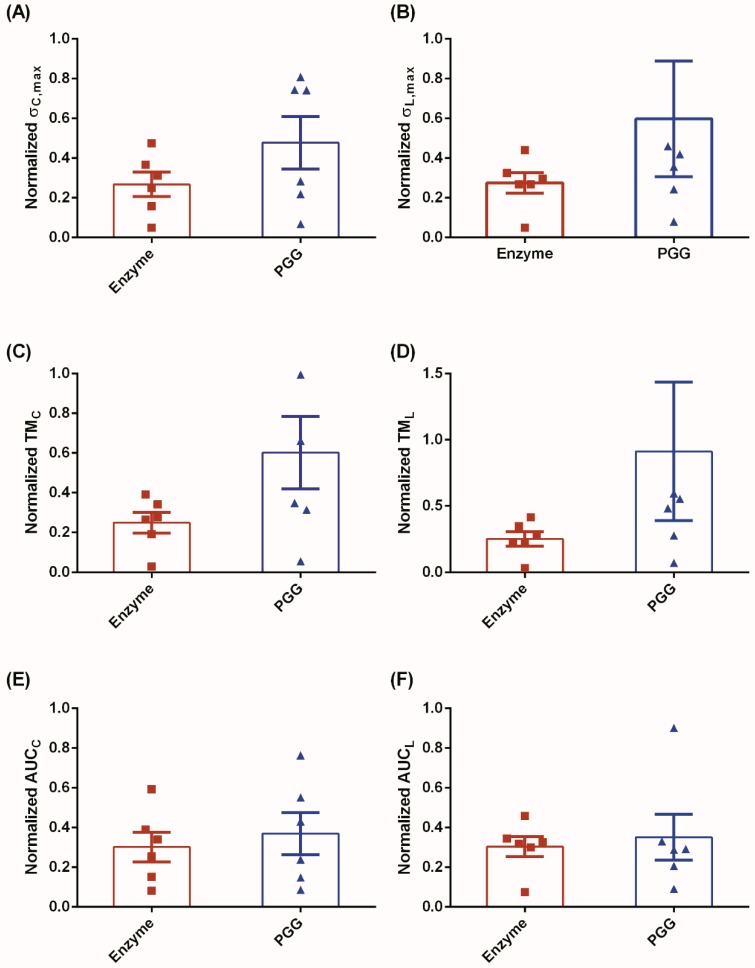
An assessment of the recovery of biomechanical properties by normalizing the enzymatic- and PGG-based biomechanical parameters to their native equivalents. We observe an increasing trend in $\sigma_{max}$ (**A**,**B**) and TM (**C**,**D**) for both circumferential and longitudinal orientations, respectively. AUC for both orientations exhibited similar but limited increase in strain energy (**E**,**F**), thereby indicating a conservation of elastic energy.

Normalized biomechanical parameters were calculated for the E and P groups to demonstrate the biomechanical recovery of the degenerated ECM owing to PGG treatment. Figure 5 shows these parameters for both tissue orientations. For the circumferential orientation, normalized $\sigma_{max}$ and TM were found to be 43.8% and 58.6% greater for P group than the E group, respectively (Figure 5A,C). However, the normalized AUC exhibited a minimal increase of approximately 18.4% from the E group to the P group (Figure 5E). Following a similar trend, normalized $\sigma_{max}$ and normalized TM were higher by 54.0% and 72.4%, respectively, for group P vs. group E in the longitudinal direction (Figure 5B,D). A small increase, of approximately 13.6%, was observed in the normalized AUC of the P group compared to the E group in the longitudinal direction (Figure 5F). 

### 3.2. Constitutive Modeling and Finite Element Analyses 

**Figure 6:**
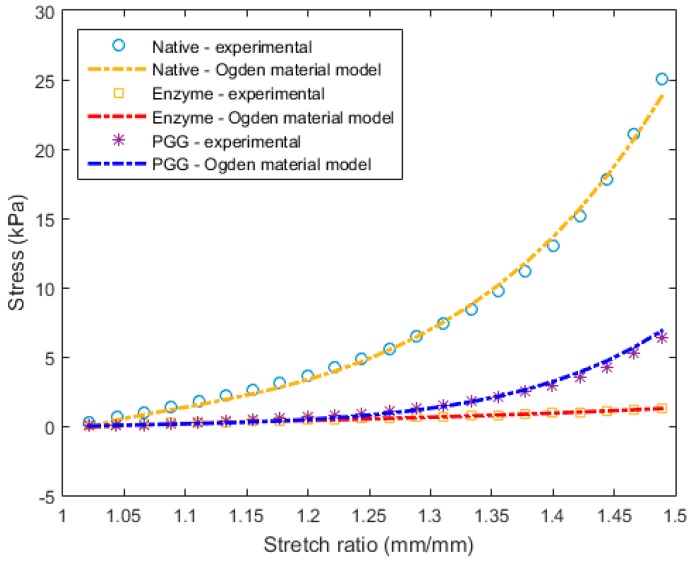
Ogden model fitting with the corresponding experimental stress–strain curves for an exemplary specimen of the native (N), enzyme (E), and PGG treatment (P) groups.

Following the Ogden constitutive relation, the phenomenological behavior was represented for each specimen of the N, E, and P groups (with exemplary stress–strain curves shown in Figure 6 and their respective material constants reported in Table 2). Good correlations (R^2^ > 0.99) between experimental and theoretical data were observed for the three experimental groups (Figure 6). 

**Figure 7:**
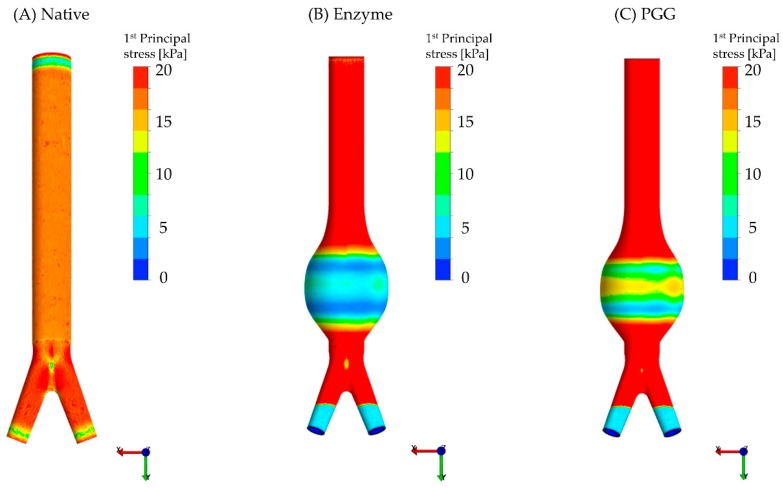
Wall stress spatial distribution for three exemplary FEA models (anterior view): (**A**) Native, (**B**) enzyme and (**C**) PGG treated. The Ogden hyperelastic constitutive material was utilized for the FEA simulations and a 100 mmHg intraluminal pressure was applied to the models. The upper limit of the stress legend was lowered to emphasize the differences across the three groups at the midsection.

The wall stress (calculated at the mid-section or sac of the geometries) obtained for each FEA model is summarized in Table 3. Figure 7 illustrates the spatial distribution of wall stress for the idealized FEA abdominal aorta models (native and AAA) based on the Ogden constitutive relations derived from stress–strain curves of the three experimental groups. Colorimetric surface plots of the wall stress in the normal aorta shows a uniform stress distribution until the aortic bifurcation (Figure 7A). Similar to the experimental data, the FEA models reveal that the maximum wall stress was in the order of N > P > E (35 ± 4.0 kPa vs. 16 ± 0.5 vs. 13 ± 1.0 kPa; *p* = 0.0002). The E and P models exhibited maximum wall stresses that were, respectively, 62.6% and 53.7% lower than the N models (Table 3). The average and minimum wall stresses at the sac region of the PGG-treated model were 1.3 and 1.7 times greater than the enzyme-treated model, respectively. However, these stresses of the PGG-treated model were almost 2.6 and 4 times lower than the native model stresses, respectively. 

## 4. Discussion

This investigation is a “proof-of-concept” contribution that highlights the beneficial crosslinking properties of PGG—specifically with respect to the degenerated arterial ECM, which is a common finding in AAA. Experimentally, we infer that PGG leads to crosslinking between the ECM proteins that improve the biomechanical strength of enzymatically degraded tissues in vitro (Table 1). To simulate the potential application of this finding, idealized finite element models were created to estimate changes in the stress build up on the aneurysmal wall (due to enzymatic damage) with and without PGG treatment (fibrillar crosslinking). The application of PGG after enzymatic degradation yielded some degree of biomechanical recovery—both experimentally and computationally (Figure 4 and Figure 5 and Table 2 and Table 3). The computational models were utilized to demonstrate changes in stress distribution owing to geometry (e.g., an aneurysmal expansion) and the effect of the constitutive material model under simulated intraluminal pressure. The primary contributions of this work are the quantification of the biomechanical restoration potential of PGG and the inference of this finding on the binding of PGG to the arterial ECM. 

### 4.1. Biomechanical Restoration Potential of PGG

The three types of tissue specimens in this study were used to represent a healthy aorta, an aneurysm pathology, and a PGG-treated aneurysmal condition, respectively. By using a mixture of enzymes (collagenase and elastase), we successfully compromised the porcine abdominal aorta ECM integrity (see Supplementary Material), which was evident by its reduced biomechanical strength (Figure 4A–F and Table 1). An hour-long digestion of arterial tissue, similar to Gundiah et al. [30], was sufficient to reduce the structural integrity of elastin and collagen. In this process, the aorta may have become more permeable to PGG influx (see Supplementary Material). The stresses in the PGG group were higher than in the enzyme-digested group, but lower than in the native group (Table 1). For clarification, the increased stresses observed in the PGG group did not yield a “stiffening” of the aorta. 

AAA rupture typically occurs when the ECM fiber distribution is altered [4,5], which results in increased stresses that exceed the strength of the diseased arterial wall. In addition, the localized concentration of stresses has been postulated as one of the primary causes of AAA rupture [1]. Noteworthy is that there was an increase in stiffness of the PGG-treated specimens (compared to the enzyme-treated specimens), but not comparable to the stiffness of the native aorta specimens (Figure 4, Figure 5 and Figure 7). The FEA results showed an average wall stress along the dilated portion of the PGG-treated aneurysmal model that was nearly 1.7 times greater than the enzymatic model, and the average stresses for both models were significantly less (nearly 2.6 times and 3.2 times, respectively) than the native model (Figure 7 and Table 3). From the experimental data, maximum stresses in the circumferential and longitudinal directions were found to decrease for the E group compared to the N group (by 77.1% and 73.3%, respectively) owing to the enzymatic cleavage of the native elastin and collagen crosslinks (Figure 4A,B). Following PGG treatment, possibly due to PGG crosslinking activity, the maximum stress for group P increased by 1.4 times in the circumferential direction and 1.6 times in the longitudinal direction compared to the E group. A similar outcome was obtained for the tensile modulus; however, only the increase in circumferential TM was significant for the PGG-treated group (almost 1.8 times greater than the enzyme digested group; Figure 4C,D). Isenberg et al. [49] reported that porcine ascending aorta specimens, treated with 0.15% PGG solution for four days, exhibited a reduced distensibility (or elastic modulus) compared to their untreated native counterparts undergoing uniaxial tension. They also suggest that this reduction in distensibility is a result of the PGG-elastin binding mechanism. However, their work does not discuss the interaction of collagen with PGG, which is one of the proline-rich compounds that selectively binds with this polyphenol [14,50]. Although our results exhibit a similar trend in elastic moduli (Figure 5C,D), they are not directly comparable to the study by Isenberg and co-authors. For example, our specimens underwent (i) biaxial tension and (ii) enzymatic degradation prior to PGG treatment (12 h at 4 °C) in contrast to Isenberg et al.’s direct PGG treatment for a longer period (four days; no temperature reported). 

Native aortic tissue exhibits anisotropic mechanical behavior due to the circumferentially oriented elastin and collagen network. We observed a non-significant anisotropy in the three experimental groups. Upon elastase treatment, arterial tissues lose some of its anisotropic characteristics [30] and concordantly, the collagen fiber arrangement is also disrupted [29]. Within 6 h of enzyme treatment, there is pronounced softening of the mechanical characteristics [47] (similar to Figure 4A–F and Table 1) and by 96 h the arterial specimens behave as a collagen-scaffold-type material [51]. The cleavage of ECM fibers by elastase and collagenase exposes some of the hydrophobic cores in the elastin fiber network. These exposed hydrophobic sites or residues are favorable for PGG attachment [17,49], as this polyphenolic compound is known to “lock” or affix the orientation of fibrous structural proteins resulting in reduced residual stresses in arteries [49]. We infer that the restoration of structural integrity and improvement of the degenerated ECM’s biomechanical characteristics (Figure 4A–F and Table 1), originates from the intricate hydrophobic bonds forged by PGG [13,52,53] that specifically crosslink arterial elastin and collagen [14,17]. 

Under typical in vivo intraluminal loads, ECM fibers in blood vessels are engaged and assumed to gradually straighten out from their “crimped” state. With increasing age (or pathology), arterial fiber arrangement is altered, leading to a change in the overall strain energy, and progressively toward a stiffened arterial matrix [54]. In our study, enzymatic and PGG treatments produced transitional change in the native microstructural fiber architecture of the porcine abdominal aorta. The strain energy (AUC) of the PGG-treated specimens exhibited some degree of recovery in both tissue directions, although it was insignificant compared to $\sigma_{max}$ or TM (Figure 5A–E). The experimental data was well represented by the hyperelastic Ogden model (Equation (6)) [41,42]; the suitability of the Ogden hyperelastic model for blood vessel mechanics is not uncommon [42,43,55]. Due to its dependence on large strain behavior, the Ogden phenomenological model fit the biaxial stress–strain curves across the three groups alike (Figure 6). To the best of our knowledge, this is the first study that compares the computational biomechanical analysis of native, enzyme-degraded, and PGG-treated abdominal aortic tissues (Figure 7A–C). 

The use of computational models with idealized geometries allowed us to simulate potential changes in wall stress distribution due to ECM modifications—either due to a pathological or a regenerative condition. FEA simulations revealed the substantial effect of the abdominal aorta geometry and the constitutive material properties on the wall stress distributions (Figure 7A–C). In addition, we noted a 62.6% and 53.6% reduction in maximum wall stress at the AAA sac of the enzyme and PGG models, respectively, compared to the native ones. The idealized native and aneurysmal models utilized in our study were similar to those of Azar et al. [44]; however, the material parameters for our FEA study were derived directly from the biaxial experiments for all native, enzyme, and PGG-treated specimens. Similar to [44], the wall stress distribution of the native aorta model was uniform until the aortic bifurcation (Figure 7A). However, the stresses reported in [44] are higher than those of our study (average wall stress—110 kPa vs. 26 kPa; maximum wall stress—760 kPa vs. 35 kPa). The intraluminal pressure applied in our FEA simulations was approximately 20 mmHg less than that of [44], which could account for the differences in the wall stresses, in addition to the different constitutive material model. Strain and stress distributions in idealized AAA geometries using the Ogden material model are presented in [43]. The stress distributions in the sac region of these geometries are similar to our models as the maximum stress is found at the bulge (as in Figure 7). They have reported a maximum von Mises stress of 135 kPa, which is less than that reported in [44], but greater than our maximum Cauchy stress, likely due to the different materials used and the wall thickness of the models. Niestrawska et al. [56] also used an idealized geometry with uniform wall thickness. They report on the maximum circumferential and longitudinal Cauchy stresses at the maximum AAA diameter for three different assumptions of fiber dispersions: non-rotationally symmetric dispersion, transversely isotropic dispersion, and isotropic. Although we have used a different constitutive equation that does not include fiber orientation, the location of the maximum stress at the AAA sac matches that of [56]. 

While the uniform wall thickness of our AAA models (groups E and P) was half of the native model, the maximum wall stress of the N group was 2.7 and 2.2 times greater than the E and P groups, respectively, due to the difference in their corresponding material properties (Table 3). Similarly, the average and minimum wall stresses of the E group (3.3 times and 6.4 times, respectively) and P group (2.5 times and 4.3 times, respectively) were lower than the N group stresses. A change in the biomechanical properties, due to PGG crosslinking, can be evidently inferred from the exhibited stress recovery in the PGG FEA models. The in silico models (similar to the experimental data) showed an increase in wall stress for the PGG-treated AAA compared to the enzymatically digested AAA. However, they also exhibited lower stresses than the native abdominal aorta (Table 3). 

### 4.2. Binding of PGG to Degenerated Arterial ECM

PGG attaches to hydrophobic portions of the proteins by surface adsorption [16], selectively binding to the elastic lamellae [17]. The disrupted aneurysmal ECM could favor the permeation of this polyphenol. Other than ECM stability, the successful inhibition of aneurysmal growth by PGG in AAA rat CaCl_2_ models in vivo is likely due to (i) its ability to be a radical scavenger [12,13,14], (ii) lower inflammatory responses [12,17,21,57,58,59], (iii) acting as a calcium antagonist (blocks inositol 1,4,5-trisphosphate receptors) [60], and (iv) reducing MMP activity [18,19,20,21,58]. In general, PGG has been shown to be less toxic than tannic acid- and glutderaldehyde-based treatments [49]. While the present work in some measure replicates the periadventitial [17,18,19] or intraluminal routes [22] of PGG administration for aneurysm suppression, our goal was to quantify the biomechanical characterization of the PGG-ECM protein binding. In rodent AAA studies, with the exception of Isenburg et al. [17] and Thirugnanasambandam et al. [21], the administration of PGG to the abdominal aorta was systemic and the aneurysm inducing-CaCl_2_ injury was created periadventitially [18,19]. Conversely, Kloster et al. [22] applied PGG intraluminally to their porcine animal model, after a combined application of elastase and balloon based-mechanical expansion of the abdominal aorta. However, it is uncertain if PGG works better with CaCl_2_‒ or elastase-based AAA models. Binding of polyphenols with proline-rich proteins is favorable for pH in the range 3.8–6.0 [50], which potentially makes it difficult for PGG to be transported and distributed through circulatory routes (blood pH is 7.4). This may be a possible explanation for why most AAA animal studies are based on a localized (i.e., not systemic) PGG administration. 

The concentration of PGG utilized in our in vitro investigation (0.6 mg/mL), was relatively higher than in the previously reported rodent studies that also utilize this unique polyphenol to prevent pathological aortic dilation [17,18,19,21]. However, it was lower than the in vivo PGG-saline formulations used by Kloster et al. (0.6 vs. 2.5 or 5 mg/mL) [22]. This high concentration reported in [21] is a possible explanation for their claim of “stiffened arterial system” as a potential side effect of PGG treatment. Moreover, there is a known difference in polyphenol activity due to variation in in vivo vs. in vitro experimental settings. Mechanistically, PGG binds to the enzyme cleaved sites of the arterial ECM and the crosslink formation between the hydrophobic cores of the elastin helps in the stabilization of the elastic lamellae. This has shown to suppress any further aortic dilation in rat CaCl_2_ aneurysm models [17,18,19,21]. Our study supports the fact that the change in aortic biomechanical properties is a direct result of this hydrophobic binding of PGG with proline-rich proteins such as elastin and collagen, even though these arterial ECM proteins were partially degraded or cleaved by a collagenase and elastase enzyme treatment. PGG has shown to lower the biomechanical stresses in rodent aneurysm models [21]; however, it is unclear if the increased wall stress, as experienced in all aneurysm models compared to native abdominal aortas, can lead to reversal of the PGG-protein binding behavior. Furthermore, it is unclear if PGG binding efficiency is affected by the degree of arterial ECM damage. 

### 4.3. Limitations

Our in vitro study has several limitations and does not completely replicate the periadventitial [17,18,19] or intraluminal routes [22] of PGG administration, so a direct comparison with known elastase AAA models is not possible. Further, the simulated aneurysmal matrix (group E) is not an exact replica of the complex ECM degradation observed in human AAA [7,34,61]. The shear components of deformation were considered negligible during the biaxial tensile experiments, similar to a previous study of the human abdominal aorta by Vande Geest et al. [37] Nevertheless, shear calculations from planar biaxial testing of soft tissues are a complex and controversial topic [38,62,63,64]. Biological tissue specimens begin degrading within a few hours of incubation in saline at room temperature (21–24 °C). It was challenging to maintain the porcine abdominal aortic specimens in a PGG solution at 37 °C for days or weeks, due to the amount of bacterial growth and its associated tissue deterioration that would take place in the solution. Inclusion of other chemicals, such as anti-bacterial agents in the PGG solution, may interfere with the overall chemical reactions and could possibly delay the reaction potential of the polyphenolic compound. In addition, we would like to clarify that enzyme treated arterial specimens underwent more rapid degradation compared to the native specimens. Even after thorough saline washes, the enzymatic degradation of the extracellular matrix continues in vitro (as it is possible for the enzymes to permeate the arterial tissue and continue the degradation process) and this degradation is maximum at body temperature (37 °C). As reported by Gundiah et al. [30], one hour of enzyme digestion is sufficient to yield significant changes in the biomechanics in vitro. At 37 °C, the remnant enzymes in the arterial tissue matrix could have further damaged the structural proteins and led to an altered biomechanical state. Therefore, specimen incubation with PGG at 37 °C, being ideal testing conditions, could have produced more arterial matrix damage than expected in vivo. Further, we found no structural differences between specimens that underwent 12 h vs. 48 h of incubation with PGG solution. Consequently, to minimize changes in the tissue microstructure due to incubation at 37 °C, we opted for 12-hour PGG treatments at 4 °C. 

The permeation of PGG in the native arterial ECM is a largely unexplored matter. For example, the permeability of the aneurysmal ECM is likely affected by the poroelastic properties of intraluminal thrombus, and/or potential biophysical interaction of PGG with the thrombus, thereby leading to several unknown queries [9,10,11,61]. Our computational models are also subject to several important limitations. We did not use a multilayer geometry or a multilayer constitutive material model (e.g., an arterial wall composed of an adventitia, media, and endothelium). Although the native and aneurysm materials show orthotropic behavior, we have implemented a first-order isotropic material model. Using a transversely isotropic (or orthotropic) or a multilayer Holzapfel–Gasser–Ogden model may improve the accuracy of the stress estimations of the present work. The FEA models lacked subject-specificity, although the use of idealized geometries in lieu of non-invasive imaging is properly justified. The intraluminal pressure loading for the FEA simulations was assumed static and spatially homogenous rather than pulsatile. The interaction between blood flow and the vessel wall was also ignored. The wall in the FEA models was impermeable and had a uniform thickness. Many of the limitations in the computational models are mitigated by the used of idealized geometries, acknowledging that the goal of the FEA simulations was to analyze the effect of constitutive material properties while maintaining the abdominal aorta geometry as a control. 

## 5. Conclusions

Due to the absence of adequate non-surgical treatment options for AAA, one possible alternative is to translate the novel PGG-based treatment from the established rodent models to prospective large animal models, and ultimately to clinical trials. In a clinical setting, a PGG-based treatment would be aimed at preventing the progressive increase in aneurysm size and the eventual rupture of the abdominal aorta. From the present work, we can infer that PGG treatment of enzyme-digested porcine aortas leads to stabilization of the arterial ECM and restores some of the tissues’ mechanical characteristics. Future investigations will focus on the tissue microstructural changes that may occur due to PGG treatment and the potential translation of this work toward an in vivo application. 

## Figures and Tables

**Table 1:** Biomechanical parameters calculated from biaxial tensile testing of porcine abdominal aorta specimens for the three experimental groups (native (N), enzyme-treated (collagenase and elastase) (E), and pentagalloyl glucose (PGG) (P)). Values are reported as mean ± SEM.

Testing Protocol	Biomechanical Parameters (kPa)	N	E	P	*p*-Value
$\lambda_{C}:\lambda_{L}$ = 1:1	$\sigma_{C,max}$	43.78 ± 14.18	10.03 ± 2.66	13.85 ± 3.02	0.0226 ^a^
	$\sigma_{L,max}$	33.89 ± 8.98	9.04 ± 2.97	14.69 ± 5.88	0.0441 ^a^
	TM_C_	195.6 ± 58.72	46.51 ± 15.04	81.8 ± 22.76	0.0314 ^a^
	TM_L_	168.0 ± 51.53	39.75 ± 15.56	101.6 ± 50.87	n.s.
	AUC_C_	6.48 ± 2.22	1.55 ± 0.34	1.56 ± 0.26	0.0224 ^a,b^
	AUC_L_	4.77 ± 1.04	1.45 ± 0.42	1.35 ± 0.32	0.0034 ^a,b^
$\lambda_{C}:\lambda_{L}$ = 0.5:1	$\sigma_{C,max}$	7.6 ± 1.35	2.65 ± 0.54	1.51 ± 0.14	0.0004 ^a,b^
	$\sigma_{L,max}$	18.66 ± 3.78	5.71 ± 1.92	4.05 ± 1.04	0.0013 ^a,b^
	TM_C_	67.46 ± 12.94	22.19 ± 5.63	16.86 ± 2.73	0.0011 ^a,b^
	TM_L_	101.9 ± 30.18	26.11 ± 10.26	31.61 ± 12.91	0.0273 ^a^
	AUC_C_	0.63 ± 0.12	0.24 ± 0.04	0.1 ± 0.01	0.0006 ^a,b^
	AUC_L_	2.84 ± 0.41	0.93 ± 0.3	0.48 ± 0.07	<0.0001 ^a,b^
$\lambda_{C}:\lambda_{L}$ = 1:0.5	$\sigma_{C,max}$	19.84 ± 5.14	5.84 ± 1.44	3.1 ± 0.73	0.0034 ^a,b^
	$\sigma_{L,max}$	6.99 ± 2.71	2.28 ± 1.299	0.8 ± 0.08	<0.0001 ^a,b^
	TM_C_	125.3 ± 38.93	30.08 ± 9.42	22.37 ± 6.28	0.0102 ^a,b^
	TM_L_	60.95 ± 12.84	18.15 ± 4.11	7.6 ± 1.38	0.0007 ^a,b^
	AUC_C_	2.64 ± 0.57	0.91 ± 0.18	0.37 ± 0.09	0.011 ^a,b^
	AUC_L_	0.59 ± 0.08	0.19 ± 0.05	0.06 ± 0.007	<0.0001 ^a,b^

^a^ denotes significant pairwise differences across N and E groups and ^b^ denotes significant pairwise differences across N and P groups (repeated measures ANOVA—sphericity assumed). n.s.: not significant.

**Table 2:** Ogden material model constants (mean ± SEM) for the three tissue types.

Group	$m_{1}$ (kPa)	$c_{1}$ (−)
N	0.96 ± 0.025	10.06 ± 0.38
E	0.37 ± 0.06	8.53 ± 0.67
P	0.09 ± 0.03	13.57 ± 0.87

**Table 3:** Wall stress (mean ± SEM) computed in the mid-section of the FEA geometries corresponding to the three Ogden material models.

Group	Maximum Wall Stress (kPa)	Average Wall Stress (kPa)	Minimum Wall Stress (kPa)
N	35 ± 4.0	26 ± 4.0	20 ± 4.0
E	13 ± 1.0	8.0 ± 0.4	3.0 ± 0.2
P	16 ± 0.5	10 ± 0.5	5.0 ± 0.3
*p*-value	0.0002 ^ab^	0.0003 ^ab^	0.0002 ^ab^

^a^ denotes significant pairwise differences across N and E groups and ^b^ denotes significant pairwise differences across N and P groups (repeated measures ANOVA—sphericity assumed).

## Supplementary Materials

The following are available online at https://www.mdpi.com/2306-5354/6/3/58/s1, Figure S1: Histological characterization of porcine arterial specimens from native (A), enzyme treated (B), and PGG treated (C) aortic specimens. Specimens were stained with Verhoeff–Van Gieson stain (VVG) for identification of elastin fibers. Images were taken at 10× magnification using a Leica^®^ microscope (Buffalo Grove, IL, USA); the scale bar size is 297.5 µm for the three frames.
