# Supplementary material for: Biomechanical Restoration Potential of Pentagalloyl Glucose after Arterial Extracellular Matrix Degeneration
Source: Bioengineering (Basel). 2019 Jul 3;6(3):58. doi: 10.3390/bioengineering6030058 (PMC6783915; doi:10.3390/bioengineering6030058)
Supplement: Supplementary file 1 [file bioengineering-06-00058-s001.pdf]

Supplementary Material

The histological analysis of the porcine abdominal aorta specimens utilized in this study was performed as follows:

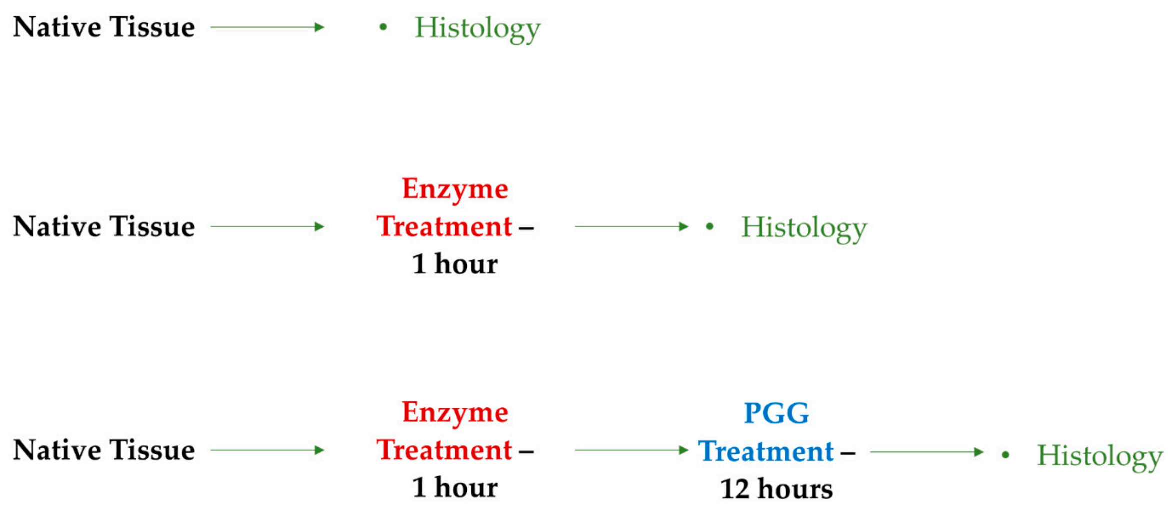

Intact aorta rings from each of the aforementioned groups were fixed with 10% neutral buffered formalin solution for at least 48 hours. After fixation, the specimens were embedded in paraffin wax and tissue blocks were generated for sectioning and further processing. Five-micron thick sections of the tissue embedded paraffin blocks were then sectioned with a microtome and transferred on a glass slide for routine histological Verhoeff–Van Gieson (VVG) staining. The images of VVG stained aorta sections are shown in Figure S1.

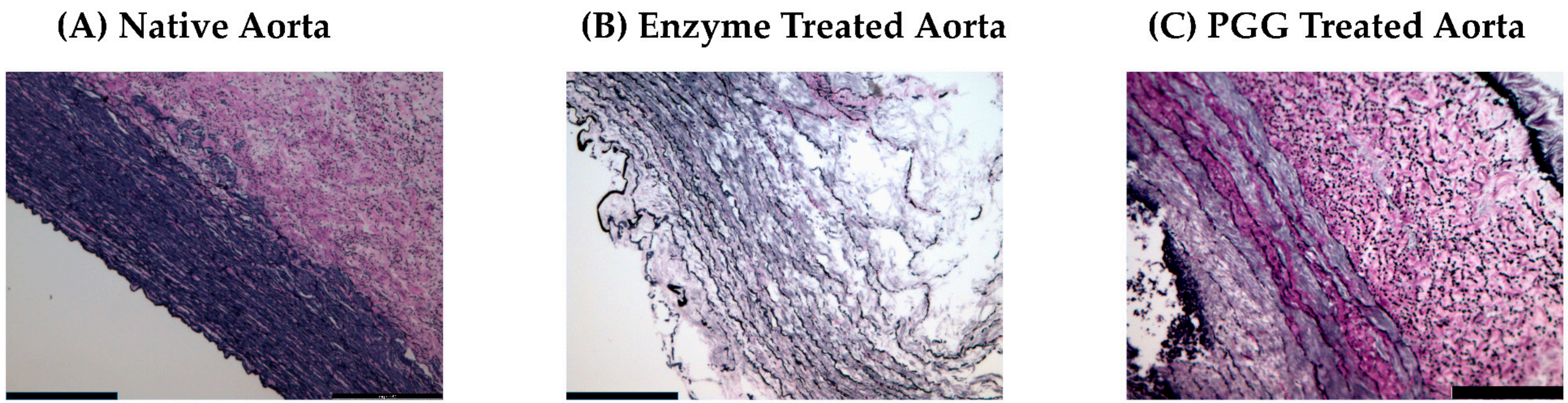

Figure S1. Histological characterization of porcine arterial specimens from native (**A**), enzyme treated (**B**), and PGG treated (**C**) aortic specimens. Specimens were stained with Verhoeff–Van Gieson stain (VVG) for identification of elastin fibers. Images were taken at 10× magnification using a Leica® microscope (Buffalo Grove, IL, USA); the scale bar size is 297.5 µm for the three frames.

VVG stains elastin fibers black and non-elastin components pink. Qualitative assessment via histology revealed that enzyme-treated specimens exhibited fragmentation and sparse fiber distribution [see Figure S1(B)] compared to the more compact fiber arrangement in the native specimens [see Figure S1(A)]. PGG is known to react with the iron chloride in the VVG staining reagents and turn black [3]. There is no specific tissue based stain for PGG (other than the general reactivity of tannic compounds like PGG with ferric chloride [3,4]) that can delineate its specific distribution in our specimens. Hence, it is possible that dense black bands on the luminal and adventitial sides could be the remnants of PGG that did not permeate the tissue completely [see Figure S1(C)]. In addition, there is noted presence of black deposits throughout the adventitial side of the porcine arterial tissues, which could also indicate some degree of PGG diffusion.
